# Clinical Effects of Integrated Traditional Chinese and Western Medicine in Treating Severe Preeclampsia and Its Influence on Maternal and Infant Outcomes after Cesarean Section under Combined Lumbar and Epidural Anesthesia

**DOI:** 10.1155/2021/6366914

**Published:** 2021-11-08

**Authors:** Xiaolu Wang, Wenpeng Wei, Yanyan Qi, Lihua Dong, Yun Zhang

**Affiliations:** ^1^Department of Obstetrics, Yantaishan Hospital, Yantai 264000, China; ^2^Department of Anesthesiology, Qingdao Eighth People's Hospital, Qingdao 266000, China; ^3^PIVAS, Affiliated Qingdao Central Hospital, Qingdao University, Qingdao 266000, China; ^4^Department of Obstetrics and Gynecology, Zhangqiu Maternity and Child Care Hospital, Jinan 250200, China; ^5^Department of Anesthesiology, Jinan Municipal Hospital of Traditional Chinese Medicine, Jinan 250012, China

## Abstract

**Objective:**

This study is aimed to observe the clinical effects of integrated traditional Chinese and Western medicine in treating severe preeclampsia (SPE) and its effects on maternal and infant outcomes after cesarean section under combined lumbar and epidural anesthesia.

**Method:**

One hundred and sixty-six pregnant women with SPE were randomly divided into an experimental group and control group, with 83 cases in each group. The control group was given conventional treatments such as magnesium sulfate, and the experimental group received self-made traditional Chinese medicine decoction for oral administration.

**Results:**

The total clinical effective rate of treatment in the experimental group was significantly higher than that in the control group. After treatment, the systolic blood pressure (SBP), diastolic blood pressure (DBP), mean arterial pressure (MAP), and 24 h proteinuria (24 h PRO) levels of the experimental group were significantly lower than those of the control group. After cesarean section (c-section) under combined lumbar and epidural anesthesia, there were statistically significant differences in placental abruption, uterine weakness, fetal intrauterine distress, and neonatal asphyxia in the experimental group, while there were no significant differences in oligohydramnios. After treatment, the contents of inflammatory factors in both groups decreased, and the decrease was more prominent in the experimental group. After treatment, the levels of blood urea nitrogen (BUN), serum creatinine (Scr), and albumin (Alb) and *β*2 microglobulin (*β*2-MG) of the two groups of patients decreased, and the levels of them in the experimental group decreased. After treatment, the levels of superoxide dismutase (SOD) and glutathione peroxidase (GSH-Px) in the two groups increased. However, the levels of malondialdehyde (MDA), lipid peroxide (LPO), and advanced oxidation protein products (AOPP) all reduced, and the increase or decrease in the experimental group was more prominent.

**Conclusion:**

The combination of traditional Chinese and Western medicine can reduce the blood pressure of a patient with SPE. After the combined spinal-epidural anesthesia and cesarean section, it can significantly improve the maternal and infant outcomes and renal function, reduce inflammatory factors levels and body oxidative stress, and increase the activities of antioxidant enzymes.

## 1. Introduction

Severe preeclampsia (SPE) is an idiopathic condition during pregnancy with persistently elevated hypertension and proteinuria (PRO) after 20 weeks of gestation period. Compared with mild-to-moderate preeclampsia, SPE patients often have significantly higher blood pressure and can even have significant symptoms such as persistent headache or upper abdominal pain [1]. SPE is a particular type of pregnancy-induced hypertension, with early onset, rapid progression, many complications, and poor perinatal prognosis, which can cause serious harm to maternal and infant health and even endanger their lives [2]. Numerous studies have shown that SPE can increase the long-term risk of cardiovascular and cerebrovascular diseases, kidney diseases, and diabetes for the mother and fetus [3]. Cesarean sections (c-sections), which can quickly separate the fetus from the adverse intrauterine environment and stop the vicious cycle between mother and fetus, are mainly used for the termination of pregnancy in patients with SPE [4]. Different anesthesia methods used in c-sections for women with SPE also have different effects on maternal hemodynamics [5]. Combination of lumbar and epidural block anesthesia is effective and widely used in normal c-sections, especially emergency c-sections [6]. However, patients in the early stage still need to rely on drug treatment. The spasmolytic and hypotensive methods are often used in clinics, while magnesium sulfate is a common spasmolytic drug, which can dilate smooth muscle and reduce blood vessel resistance and blood pressure in pregnant women [7]. However, there are individual differences in the effect of magnesium sulfate, which may lead to magnesium poisoning and poor safety [8]. According to the traditional Chinese medicine system, preeclampsia belongs to the category of “pregnancy vertigo” and “gestational vertigo” [9]. During pregnancy, the accumulation of Yin and blood, deficiency of essential qi, imbalance of Yin and Yang of liver and kidney, and disorder of qi and blood result in eclampsia [9]. Therefore, one hundred and sixty-six cases of SPE in our hospital were treated with TCM decoction with magnesium sulfate and other conventional treatment, and the outcomes were satisfactory.

## 2. Materials and Methods

### 2.1. General Information

A total of 166 pregnant women with SPE treated at Yantaishan Hospital, Yantai, Shandong, China, from April 2018 to September 2020, were selected, all of them met the relevant diagnostic criteria for SPE in China [10]. Inclusion criteria were as follows: (1) aged from 20 to 35 years; (2) all were first pregnancies; (3) single child; and (4) signed informed consent and voluntary participation in the study. Exclusion criteria were as follows: (1) patients with chronic kidney disease and history of chronic hypertension before pregnancy; (2) patients with severe heart, brain, liver, lung, kidney, and other important organ dysfunction, as well as coagulation dysfunction, blood system diseases; and (3) patients with other malignant tumors, hemorrhagic diseases, drug allergies, gestational diabetes, etc. Patients were randomly divided into an experimental group and control group, eighty-three cases in each group. The experimental group was 21–33 years old, with an average of 27.2 ± 3.4 years old; gestational age ranged from 24 to 33 weeks, mean 29.6 ± 2.7 weeks; and body mass index (BMI) was 21.4∼28.9 g/m^2^, with an average of 25.3 ± 2.7 g/m^2^. The control group was 23–32 years old, with an average of 27.6 ± 3.6 years old; gestational age ranged from 25 to 33 weeks, with an average of 29.3 ± 2.8 weeks; and BMI was 21.7–29.4 g/m^2^, mean 25.8 ± 2.9 g/m^2^. There was no significant difference between the two groups of general information (*P* > 0.05), and they were comparable. This study was approved by the Ethics Committee of Yantaishan Hospital, Yantai, Shandong, China.

### 2.2. Diagnostic Criteria

Western medicine diagnostic criteria were as follows: patients meet the criteria of “Chinese Obstetrics and Gynecology” [11] for early-onset SPE: hypertension occurs for the first time after 20 weeks of pregnancy, that is, systolic blood pressure (SBP) ≥ 160 mmHg and/or diastolic blood pressure (DBP) ≥ 110 mmHg, accompanied by any of the following: (1) PRO ≥ 50 g/24 h, or random PRO was positive, or PRO/creatinine ≥0.3; (2) no PRO, but with heart, liver, lung, kidney and other important organs, or digestive system, blood system, nervous system and other abnormal changes, placenta-fetal involvement. TCM diagnostic criteria: patients meet the criteria for syndromes of Yin deficiency and liver vigor in “Chinese Medicine and Gynecology” [12]; dizziness, tinnitus, insomnia, blurred vision, anguish are the main symptoms, facial blushing, dry mouth and throat, feverishness in palms and soles are secondary symptoms, and red tongue, less moss, pulse a few strings.

### 2.3. Treatment Method

After admission, patients in both groups were given conventional treatment: 5 g 25% magnesium sulfate injection (Tianjin Pharmaceutical Jiaozuo Co., LTD., Approval No. H20043974) with 100 mL 10% glucose injection was administered intravenously within half an hour for spasmolysis. Then, 15 g 25% magnesium sulfate injection and 500 mL 5% glucose injection were given intravenously. At the same time, 2.5 g magnesium sulfate injection could be given intramuscularly according to the patient's blood pressure, but the total dosage of magnesium sulfate was less than 30 g, 3 times per day, 2.5 mg per time. On this basis, the experimental group was supplemented with our hospital's self-made Chinese medicine decoction. The prescriptions were uncariae ramulus cum uncis (15 g), *Salvia miltiorrhiza* (15 g), *Astragalus propinquus* (15 g), puerariae lobamle radix (15 g), eucommiae cortex (12 g), leonuri herba (12 g), ginseng radix et rhizoma (10 g), paeoniae radix alba (10 g), poria (10 g), dioscoreae rhizoma (10 g), 5 g of angelicae sinensis radix, and 3 g of glycyrrhizae radix et rhizoma. The above prescription was decocted to 200 mL, once a day, orally with warm water twice in the morning and evening. Treatment was terminated 1 day before termination of pregnancy in both groups.

### 2.4. Observation Index

(1) The clinical efficacy of the two group patients was compared [13]. Special effect: clinical symptoms disappeared completely or significantly improved, blood pressure decreased >20 mmHg (1 mmHg = 0.133 kPa), and PRO and edema symptoms disappeared. Valid: clinical symptoms improved, blood pressure decreased by 10–20 mmHg, and edema and PRO both improved significantly. Invalid: those who did not meet the above standards. The total effective rate of treatment = (special effect + valid) number of cases/total number of cases × 100%. (2) Blood pressure (SBP, DBP), mean arterial pressure (MAP), and 24-h PRO levels were compared between the two groups after treatment. (3) Serum levels of interleukin-6 (IL-6), tumor necrosis factor-*α* (TNF-*α*), C-reactive protein (CRP), and homocysteine (Hcy) were compared between the two groups before and after treatment. IL-6 and TNF-*α* levels were determined by an enzyme-linked immunosorbent assay (ELISA) kit. Hcy levels were determined by the circulating enzyme method, and serum CRP levels were determined by immunoturbidimetry. (4) The stress indexes of the two groups were compared before and after treatment. The activity of superoxide dismutase (SOD) was determined by the xanthine oxidase method. The activity of glutathione peroxidase (GSH-Px) was determined by the dithio-dinitrobenzoic acid method. The levels of malondialdehyde (MDA) and lipid peroxide (LPO) were determined by the thiobarbituric acid method. And the level of advanced oxidation protein products (AOPP) was determined by the chloramine colorimetric method. (5) The renal function of the two groups was compared before and after treatment. Serum creatinine (SCr) and blood urea nitrogen (BUN) were detected by immunoturbidimetry, and albumin (Alb) and *β*2 microglobulin (*β*2-MG) were detected by radioimmunoassay. (6) The incidence of adverse pregnancy outcomes was compared between the two groups. Before cesarean section under combined lumbar and epidural anesthesia, routine fasting was 8 h, and drugs such as hibernation mixture and magnesium preparation were given within 6 h before operation. The parturient was placed in a standard right decubitus position and routinely disinfected. Intervertebral needle insertion was selected between L3 and 4. 10 mg bupivacaine injection (Shanghai Harvest Pharmaceutical Co., LTD) was injected into the subarachnoid space. Placental abruption, uterine asthenia, fetal distress, neonatal asphyxia, and oligohydramnios were recorded.

### 2.5. Statistical Analysis

SPSS 20.0 software was used for statistical analysis of the data obtained in this study. The measurement data were expressed as $\bar{x}\pm s$ and the *T*-test was adopted. The count data were expressed in percentage , and the *χ*^2^ test was used. *P* < 0.05 was considered statistically significant.

## 3. Results

### 3.1. Comparison of Clinical Efficacy between the Two Groups of Patients

The total clinical effective rate of treatment in the experimental group was 90.36% and in the control group was 71.08% (Table 1). The difference between the two groups was statistically significant (*χ*^2^ = 13.267, *P*=0.001) (Table 1).

### 3.2. Comparison of Blood Pressure, MAP, and 24 h PRO Content between the Two Groups of Patients after Treatment

**Figure 1:**
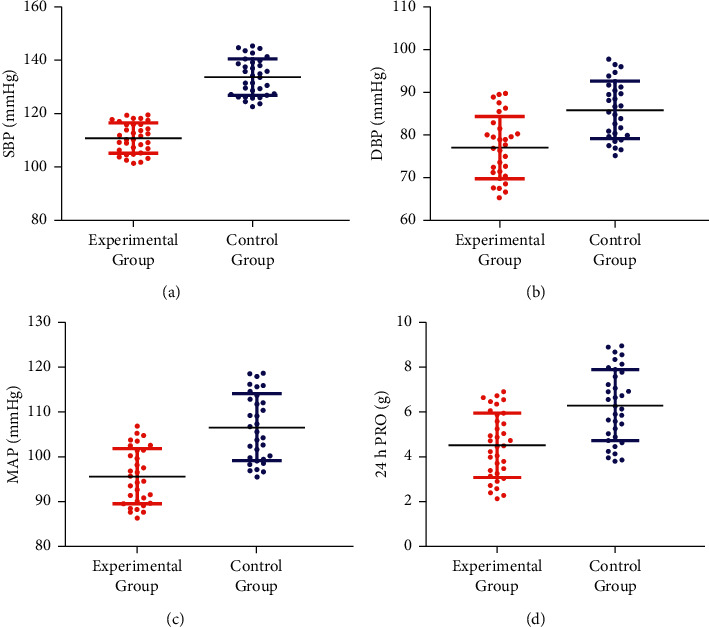
Comparison of blood pressure, MAP, and 24 h PRO content between the two groups of patients after treatment. (a) The comparison of SBP after treatment of the two groups of patients. (b) The comparison of DBP after treatment of the two groups of patients. (c) The comparison of the MAP between the two groups of patients after treatment. (d) The comparison of PRO content of the two groups of patients at 24 h after treatment.

After treatment, the SBP, DBP, MAP, and 24 h PRO levels of the experimental group were significantly lower than those of the control group (Figure 1).

### 3.3. Comparison of Serum Inflammatory Factor Levels before and after Treatment between the Two Groups

**Figure 2:**
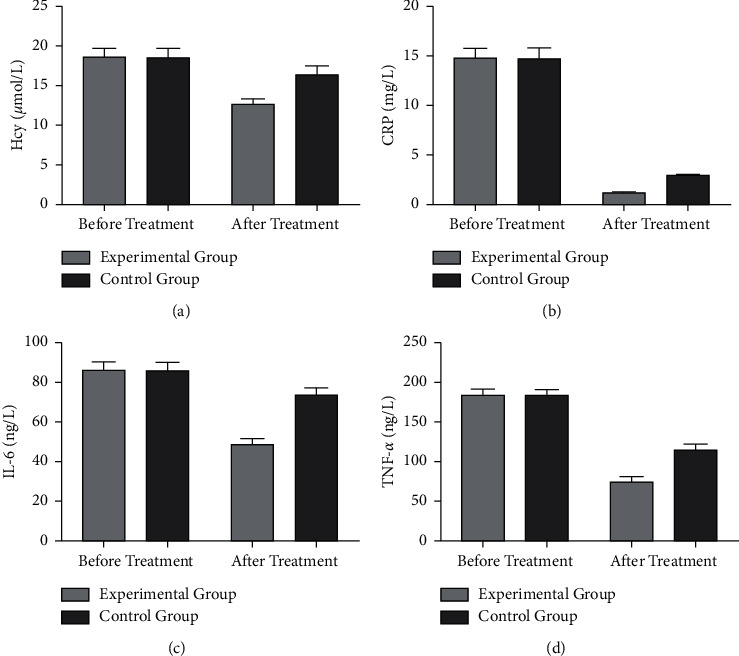
Comparison of serum inflammatory factor levels before and after treatment between the two groups. (a) The comparison of Hcy levels between the two groups of patients before and after treatment. (b) The comparison of CRP levels between the two groups of patients before and after treatment. (c) The comparison of IL-6 levels before and after treatment in the two groups of patients. (d) The comparison of TNF-*α* levels before and after treatment in the two groups.

Before treatment, there was no significant difference in serum Hcy, CRP, IL-6, and TNF-*α* levels between the two groups of patients (*P* > 0.05) (Figure 2). After treatment, the levels of inflammatory factors in the two groups of patients decreased, and the decrease in the experimental group was more significant (*P* < 0.05) (Figure 2).

### 3.4. Comparison of Stress Indicators between the Two Groups of Patients before and after Treatment

Before treatment, there was no significant difference in the content of stress indicators between the two groups of patients (*P* > 0.05) (Table 2). After treatment, the levels of SOD and GSH-Px in the two groups increased, and the increase in the experimental group was more significant (*P* < 0.05) (Table 2). The levels of MDA, LPO, and AOPP in the two groups of patients decreased, and the decrease in the experimental group was more significant (*P* < 0.05) (Table 2).

### 3.5. Comparison of Renal Function Indexes between the Two Groups of Patients before and after Treatment

**Figure 3:**
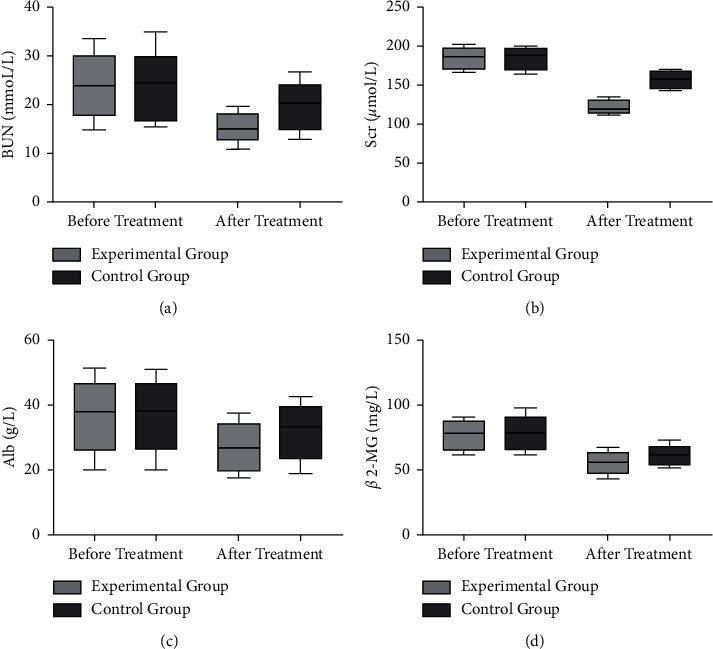
Comparison of renal function indexes of the two groups of patients before and after treatment. (a) The comparison of BUN levels between the two groups of patients before and after treatment. (b) The comparison of Scr levels between the two groups of patients before and after treatment. (c) The comparison of Alb levels between the two groups of patients before and after treatment. (d) The comparison of *β*2-MG levels before and after treatment in the two groups.

Before treatment, there was no significant difference in renal function indexes between the two groups (*P* > 0.05) (Figure 3). After treatment, the levels of BUN, Scr, Alb, and *β*2-MG of the two groups of patients decreased, and the levels of various indicators in the experimental group decreased more significantly (*P* < 0.05) (Figure 3).

### 3.6. Comparison of Maternal and Infant Outcomes between the Two Groups

The experimental group had statistically significant differences in placental abruption, uterine asthenia, fetal distress, and neonatal asphyxia (*P* < 0.05), and there was no statistically significant difference in the comparison of oligohydramnios (*P* > 0.05) (Table 3).

## 4. Discussion

SPE is a common complication of pregnancy-induced hypertension, and its pathogenesis may be related to genetics, diet, immune regulation, and other factors, resulting in elevated blood pressure and inadequate placental perfusion [14]. If the treatment is not given timely, it can lead to abortion and even pose a threat to the life safety of pregnant women and fetuses [15]. Therefore, early detection and early treatment are very important to improve outcomes in mother and infant. Magnesium sulfate is the drug of choice for the treatment of SPE, which has the effect of relieving spasm of vascular smooth muscle, inhibiting central and motor nerves and the release of acetylcholine, reducing muscle contraction, dilating vascular smooth muscle, expanding spasm of peripheral blood vessels, and reducing blood pressure [16]. However, the clinical effect of single use is not good because severe placental ischemia and hypoxia will lead to maternal endothelial dysfunction [17]. Studies have found that giving a scientific dose of magnesium sulfate for preeclampsia maternity can reduce the incidence of cerebral palsy in children [18]. However, Ugwu et al. reported that long-term application of magnesium sulfate has the risk of magnesium ion poisoning, which is not conducive to maternal and child health [19]. Therefore, searching for safe and more effective prevention and treatment measures has become an urgent medical problem in obstetrics.

TCM experts believes that the key treatment principles for SPE are nourishing Yin and tonifying kidney, calming liver, and promoting blood circulation [20]. Glycyrrhizae radix et rhizoma mixed with various medicines, *Salvia miltiorrhiza* and *Astragalus propinquus*, can promote blood circulation and remove stasis, nourish blood and tranquilizing mind, and regulate the metabolism of water and salt in the body [21]. At the same time, studies have found that *Salvia miltiorrhiza* can also effectively remove oxygen free radicals in the body and reduce or block microcirculation disorders in the body, so as to significantly improve the renal function of pregnant women with SPE [22]. Angelicae sinensis radix and leonuri herba can nourish qi and promote blood circulation; ginseng radix et rhizoma can improve the metabolic products in the blood of patients with hypertension and restore normal body function; uncariae ramulus cum uncis has the effect of calming the liver, extinguishing the wind, and reinforcing yang; paeoniae radix alba can soothe the liver and relieve pain and nourish blood for regulating menstruation; dioscoreae rhizoma can invigorate spleen-stomach and replenish qi, nourish the stomach and spleen, and strengthen yin and benefit kidney; eucommiae cortex can reinforce liver and kidney; puerarin is the main isoflavone compound of puerariae lobamle radix, which has been widely used in clinical treatment of cardiovascular diseases; poria can invigorate spleen for eliminating dampness [23–26]. Recent pharmacological studies shown that puerarin can reduce the content of endothelin and play a role in reducing blood pressure. The combination of all drugs play the effect of tonifying liver and kidney, nourishing yin and helping yang, calming wind, and relieving spasm.

According to the results of this study, the total clinical effective rate of the experimental group was obviously higher than that of the control group, indicating that the treatment of SPE by integrated traditional Chinese and Western medicine is better than that by Western medicine alone. After treatment, the contents of SOD and GSH-Px in the experimental group were notably higher than those in the control group, while the levels of MDA, LPO, and AOPP in the experimental group were dramatically lower than those in the control group, which may be related to the components of *Salvia miltiorrhiza* in the TCM decoction. It has the effect of promoting blood circulation and removing blood stasis, improving vascular endothelial function damage, enhancing antioxidant capacity of the body, and alleviating oxidative stress injury [27]. After treatment, blood pressure, MAP, and 24-h PRO content in the experimental group were markedly lower than those in the control group. In this study, puerariae lobamle radix and uncariae ramulus cum uncis have antihypertensive effects and help control blood pressure. IL-6, TNF-*α*, and CRP can cause vascular endothelial dysfunction and participate in the occurrence of hypertension during pregnancy [28, 29]. Elevated levels of Hcy can aggravate vascular endothelial cell damage and promote eclampsia. After treatment, the level of inflammatory factors in the experimental group was obviously lower than that in the control group, and the renal function was significantly better than that in the control group, indicating that the TCM decoction used in this study has the potential of nourishing Yin and kidney, can reduce the level of inflammatory factors in the body, and improve the immune function of the human body. C-section is an important means of pregnancy termination for patients with SPE. Combination of lumbar and epidural block anesthesia has the advantages of small dosage, perfect block, and quick effect, which can reach the anesthesia level required by operation in a short time, and has good analgesic effects and high safety profile [30]. In terms of maternal and infant outcomes, the experimental group was superior to the control group as a whole, suggesting that the addition of Chinese medicine decoction used in this study can significantly reduce the patients' blood pressure and the damage to the mother and fetus.

In summary, the combination of traditional Chinese and Western medicine has significant therapeutic effects in the treatment of SPE, which can decrease blood pressure, IL-6, TNF-*α*, CRP, and Hcy levels and oxidative stress and improve the body's immune function. Therefore, this therapeutic strategy can reduce the incidence of adverse pregnancy outcomes after c-section under combined lumbar and epidural anesthesia.

## Figures and Tables

**Table 1:** Comparison of clinical efficacy between the two groups of patients (*n* (%)).

Group	Cases	Special effect	Valid	Invalid	Total effective rate
Experimental	83	36 (43.37)	39 (46.99)	8 (9.64)	75 (90.36)
Control	83	19 (22.89)	40 (48.19)	24 (28.92)	59 (71.08)
*χ* ^2^					13.267
*P*					0.001

**Table 2:** Comparison of stress indicators between the two groups of patients before and after treatment ($\bar{x}\pm s$).

Indicators		Experimental	Control	*t*	*P*
SOD (U/mL)	Before treatment	83.66 ± 9.63	84.07 ± 9.73	0.264	>0.05
	After treatment	141.57 ± 6.34	113.28 ± 7.46	7.823	<0.05

GSH-Px (U/mL)	Before treatment	98.37 ± 10.84	98.72 ± 10.21	0.665	>0.05
	After treatment	133.56 ± 8.71	110.42 ± 9.02	10.234	<0.05

MDA (mmol/L)	Before treatment	9.23 ± 2.37	9.15 ± 2.64	0.456	>0.05
	After treatment	4.87 ± 1.13	7.23 ± 1.58	4.871	<0.05

LPO (nmol/L)	Before treatment	15.87 ± 3.72	15.96 ± 3.45	0.674	>0.05
	After treatment	7.34 ± 2.23	12.62 ± 2.73	6.257	<0.05

AOPP (*μ*mol/L)	Before treatment	31.36 ± 5.24	31.93 ± 5.48	0.931	>0.05
	After treatment	18.37 ± 3.66	25.89 ± 3.91	12.554	<0.05

**Table 3:** Comparison of maternal and infant outcomes between the two groups (n).

Group	Cases	Placental abruption	Oligohydramnios	Uterine asthenia	Fetal distress	Neonatal asphyxia
Experimental	83	6	4	5	4	2
Control	83	16	7	13	13	9
*χ* ^2^		6.631	1.132	5.457	10.226	11.392
*P*		<0.05	>0.05	<0.05	<0.05	<0.05

## Data Availability

The datasets used and/or analyzed during the present study are available from the corresponding author on reasonable request.
